# Endothelial Aging Associated with Oxidative Stress Can Be Modulated by a Healthy Mediterranean Diet

**DOI:** 10.3390/ijms14058869

**Published:** 2013-04-24

**Authors:** Carmen Marín, Elena M Yubero-Serrano, José López-Miranda, Francisco Pérez-Jiménez

**Affiliations:** Lipids and Atherosclerosis Unit, Maimonides Institute for Research in Biomedicina at Cordoba (IMIBIC)/Reina Sofia University Hospital/University of Cordoba and CIBER Fisiopatologia Obesidad y Nutricion (CIBERobn), Instituto de Salud Carlos III, Cordoba, 14004, Spain; E-Mails: cmhinojo@yahoo.es (C.M.); helese35@hotmail.com (E.M.Y.-S.); jlopezmir@gmail.com (J.L.-M.)

**Keywords:** aging, oxidative stress, diet, telomeres, endothelial progenitor cell

## Abstract

Aging is a condition which favors the development of atherosclerosis, which has been associated with a breakdown in repair processes that occurs in response to cell damage. The dysregulation of the biological systems associated with aging are produced partly through damage which accumulates over time. One major source of this injury is oxidative stress, which can impair biological structures and the mechanisms by which they are repaired. These mechanisms are based on the pathogenesis of endothelial dysfunction, which in turn is associated with cardiovascular disease, carcinogenesis and aging. The dependent dysfunction of aging has been correlated with a reduction in the number and/or functional activity of endothelial progenitor cells, which could hinder the repair and regeneration of the endothelium. In addition, aging, inflammation and oxidative stress are endogenous factors that cause telomere shortening, which is dependent on oxidative cell damage. Moreover, telomere length correlates with lifestyle and the consumption of a healthy diet. Thus, diseases associated with aging and age may be caused by the long-term effects of oxidative damage, which are modified by genetic and environmental factors. Considering that diet is a very important source of antioxidants, in this review we will analyze the relationship between oxidative stress, aging, and the mechanisms which may be involved in a higher survival rate and a lower incidence of the diseases associated with aging in populations which follow a healthy diet.

## 1. Introduction

Aging is defined as the natural decline in chances of survival which all species suffer with advancing age. It is expected that in Europe, by 2025, the size of the elderly population will have reached 198 million, 78.5% more than in 1975 [1]. The dysregulation of the biological systems associated with aging are produced partly through damage that accumulates over time [2]. Understanding the molecular and cellular mechanisms that underlie the aging process would provide a good strategy to address the problems presented by the aging of the world’s population.

Age-specific mortality rates from cardiovascular diseases (CVD) and strokes increase with age throughout the later years of life. Thus, the aging process is the main risk factor for the development of CVD, and is associated with alterations of the structure and function of vascular components, such as the endothelium and vascular smooth muscle cells (VSMCs), through various pathways, including oxidative stress, cell senescence and inflammation [3–5].

Among the biological structures that are progressively affected by aging, the endothelium is one of the most important because it is in charge of the regulation of vascular homeostasis by the production of nitric oxide (NO) [6]. Previous evidence has demonstrated that in the absence of other risk factors, aging *per se* causes the development of atherosclerosis. Therefore, aging could be considered as an independent factor associated with endothelial dysfunction even in the absence of other cardiovascular risk factors such as hypertension, diabetes mellitus, hypercholesterolemia, cigarette smoking or a sedentary lifestyle, as well as genetic factors [7,8].

**Figure 1:**
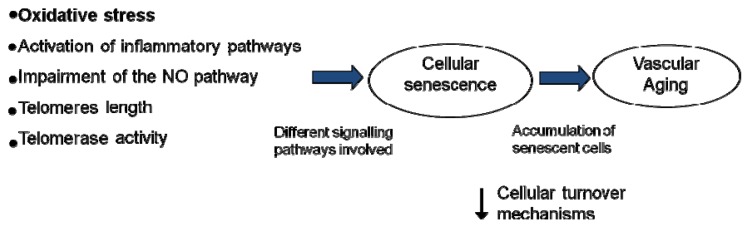
Postulated mechanisms involved in the vascular aging process. Vascular aging can be induced by different factors as oxidative stress, inflammation, an impairment of NO pathway and/or length and activity of telomerase, producing a decrease in cellular turnover mechanisms and an accumulation of senescent cells.

Several studies suggest that the impairment of the endothelial function is a progressive, multifactorial phenomenon in the elderly, with several pathophysiological mechanisms contributing to the functional deterioration of vascular endothelial cells, and this is considered as one of the main processes by which aging increases the risk of CVD and the development of atherosclerosis in humans [9–11]. In this sense, there must be a balance between the rate of cellular damage and renewal to maintain homeostasis and tissue function. Therefore, the research approaches aimed at preserving or improving the endothelial function should play a key role in the prevention of vascular diseases in the elderly. One major mechanism involved in the vascular aging process is oxidative stress [8,12]. Considerable evidence has been published indicating that increased production of reactive oxygen species (ROS) leads to endothelial dysfunction in aging both in animals [13] and in humans [14]. However, several other possible mechanisms have been postulated: impairment of the NO pathway [8,15,16], activation of inflammatory pathways [17–19], telomere length and telomerase activity [20,21], and the senescence of endothelial progenitor cells [22,23] (Figure 1). This complex process is controlled by several factors, such as changes in lifestyle, diet and physical activity, as well as drug treatments and medication [24–26] (Table 1). In this way, the use and application of these alternatives could prevent and/or treat advanced stages of endothelial dysfunction or act on the structural alterations of the vascular wall [27,28].

Greater knowledge of the molecular and cellular mechanisms involved in vascular dysfunction associated with aging would provide a better understanding with which we could develop suitable strategies, use specific targets to mitigate the effect of vascular aging, prevent cardiovascular diseases and improve the quality of life of the elderly. After that, we will analyze the mechanisms involved in vascular dysfunction related with aging and review the possible benefits of dietetic strategies with potential to promote cardiovascular health in the elderly.

For this review, we have carried out a systematic search of the Pubmed database from 1990 to February 2013. The keywords used in the Pubmed search were “aging, oxidative stress, vascular aging, diet and Mediterranean diet.” Our search strategy yielded 13,078 citations. Irrelevant papers were excluded by title and abstract reviews, which narrowed it down to 126 manuscripts. We reviewed scientific articles considering the scientific evidence that included: (1) The effect of oxidative stress on vascular aging; (2) the effect of nutrients and dietary intervention on vascular aging; (3) repairing the damage induced by oxidative stress and the inflammatory process in aging.

## 2. Vascular Aging and Oxidative Stress

The underlying mechanisms in vascular aging are complex, and involve different pathways [47]. Oxidative stress is caused by an imbalance between the production of reactive oxygen and a biological system’s ability to readily detoxify the reactive intermediates or easily repair the resulting damage. A large body of evidence indicates that oxidative stress is increased during aging, which is caused by the imbalance between ROS production and antioxidant defense capability (enzymatic and non-enzymatic antioxidants) [12]. Various hypotheses suggest that the decline in endothelial function during aging is due to an increase in superoxide anion (O_2_^−^) levels, which lead to a decrease in the availability of NO [48].

The main sources of ROS in the endothelium are composed of a variety of cell types, including VSMCs, endothelial cells and mononuclear cells. ROS include superoxide anion (O_2_^−^), hydrogen peroxide (H_2_O_2_), hydroxyl radical (OH), hypochlorous acid (HOCl), NO and peroxynitrite (ONOO^−^). The antioxidant enzyme superoxide dismutase (SOD) rapidly dismutates O_2_^−^ to H_2_O_2_, and subsequently, the H_2_O_2_ is eliminated by glutathione peroxidase (GSH-Px) and catalase and turned into water.

Aging activates the enzymes involved in ROS production, such as nicotinamide adenine dinucleotide phosphate (NADPH) oxidase, xantine oxidase, uncoupled NO synthase and cyclooxygenase and it inactivates the antioxidant system, including SOD, (GSH-Px) and catalase, leading to an increase in ROS production and a decrease in ROS degradation. However, the results obtained from different studies in which antioxidant defense levels were determined were contradictory. While some authors observed an age-related decrease in superoxide dismutase (SOD) and glutathione peroxidase (GSH-Px) activities and a decline in plasma antioxidant capacity [46], others found an increase in the activity of SOD and GSH-Px [49].

Another source for aging-induced vascular dysfunction is oxidative stress generated by mitochondria [44]. Under physiological conditions, mitochondria produce O_2_^−^ and H_2_O_2_, so that mitochondrial DNA (mtDNA) is particularly exposed to oxidative damage. During aging, the result is a reduction in the number of mitochondria and a higher expression of dysfunctional proteins, which leads to the malfunction of the respiratory chain energy, increasing O_2_^−^ production and a depletion in energy supply to cells [50].

The implication of oxidative stress in the development of vascular aging has been described in both laboratory animals [51,52] and humans [14,53]. Upregulation of pro-oxidants and downregulation of antioxidants leads to consequences in vascular remodeling by VSMCs proliferation, migration and extracellular matrix remodeling [54], generating an impairment of endothelial function, inflammation, apoptosis and senescence of endothelial cells [55].

## 3. Vascular Aging and Inflammation

Abundant experimental and clinical data show that aging is also associated with chronic low-grade inflammation. Because there is an important cross talk among inflammatory processes, generation of ROS and endothelial dysfunction [56], recent studies showed that ROS *per se* can act as molecule-signaling activating pathways which regulate inflammatory processes, including secretion of inflammatory mediators. Inflammation itself promotes cellular oxidative stress [53]. ROS interacts with different redox-sensitive transcriptional factors such as activator protein 1 (AP-1) and nuclear transcription factor-kappa B (NF-κB), increasing the gene expression of cytokines (TNFα, IL-1β and IL-6) and adhesion molecules (Intercellular Adhesion Molecule 1 (ICAM-1) and vascular cell adhesion protein 1 (VCAM-1)). An increase in inflammatory cytokine levels contributes to a pro-inflammatory status, facilitates the development of vascular dysfunction, and promotes endothelial apoptosis in aging [57]. In this regard, it is significant that caloric restriction can attenuate the altered signaling transduction of inflammatory processes, which are mediated through NF-κB and AP-1, and endothelial activation in aged rats [42,43,58]. Furthermore, it has numerous beneficial effects on the aging cardiovascular system by a reduction in the inflammatory process in the vasculature and heart, and it could protect against the development of arterial stiffness with increasing aging [32].

## 4. Vascular Aging and Alteration of the NO Pathway

Nitrosative stress is defined as the ratio of nitrosants and antioxidants similar to oxidative stress, but with the additional involvement of reactive nitrogen species. Oxidative/nitrosative stress represent the imbalance in the production and the elimination of reactive oxygen and nitrogen species. Many functions of the vascular endothelium are modulated by NO, which is able to confer vasoprotective and cardioprotective effects, including the production of smooth muscle relaxation [10]; inhibition of platelet activation and adhesion to the surface of the endothelium [59]; disruption of synthesis and expression of cytokines and cell adhesion molecules [47]; preservation of endothelial progenitor cell (EPC) function; and regulation of tissue energy metabolism. NO is synthesized from l-arginine by the enzyme NO synthase (NOS). There are three known NOS isoenzymes: the constitutive endothelial (eNOS) and neuronal (nNOS) isoforms, which produce the NO involved in regulatory pathways, and the inducible (iNOS) NOS isoform, which produces an uncontrolled NO synthesis, related with inflammatory responses. As we mentioned previously, the reduction of NO availability caused by excess ROS production is a major cause of endothelial dysfunction in aging, altering vascular homeostasis [60,61]. This reduced NO production may be controlled by several mechanisms: (1) a deficiency in NOS substrates and cofactors; (2) the presence of endogenous eNOS inhibitors; and (3) a lower expression and/or activity of eNOS.

### 4.1. Deficiency in NOS Substrates and Cofactors

**Figure 2:**
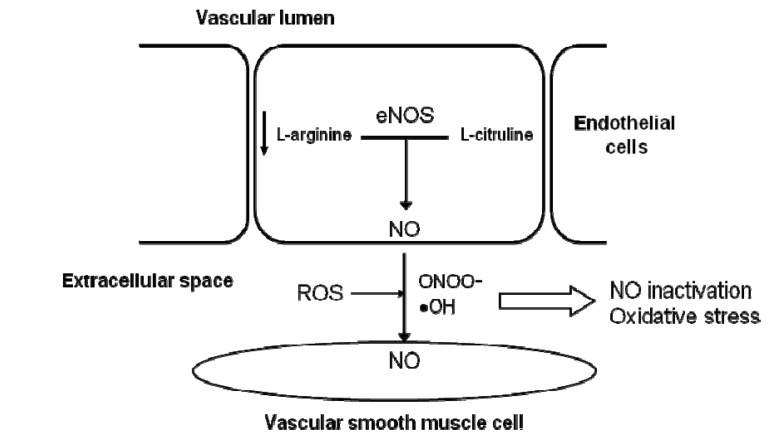
Mechanisms responsible for a low NO availability during aging. The oxidation of L-arginine produces NO and l-citruline by the endothelial nitric oxide synthase (eNOS). The presence of low levels of l-arginine contribute to a low NO availability during aging.

One of the mechanisms responsible for a low NO availability during aging is the presence of a lower concentration of l-arginine, which is used as eNOS substrate to produce NO in endothelial cells. The oxidation of l-arginine produces NO and l-citruline by endothelial nitric oxide synthase (eNOS) (Figure 2) [62]. The contribution of an increased expression and/or activity of arginase, the enzyme that degrades l-arginine, to age-related endothelial dysfunction could explain a decrease in substrate availability for eNOS and the consequent reduction of NO synthesis [63]. This is not the only mechanism that leads to impaired NO production, but it also contributes to an enhanced production of ROS by NOS (Figure 2). Although the role of arginase in endothelial dysfunction in elderly patients requires further investigation, some studies suggest that this alteration described in animals might also appear in humans [64].

Tetrahydrobiopterin (BH_4_), a cofactor essential for NOS activity, is an allosteric factor in the coupling of the oxidase and reductase domains of eNOS [65]. The regulation of eNOS by the oxidation of the cofactor BH4 is an important contributor to endothelial dysfunction. ROS induces eNOS uncoupling, altering its catalytic activity. eNOS uncoupling produces ROS rather than NO, and therefore, when BH4 is limited or under oxidative stress conditions, this cofactor produces superoxide, leading to peroxinitrite [65,66]. Several studies have shown that the administration of BH4 to older adults causes a selective improvement in endothelial vasorelaxation, demonstrating that BH4 potentially leads to eNOS recoupling in aged human vasculature [67].

### 4.2. Presence of Endogenous eNOS Inhibitors

An endogenous l-arginine analog, asymmetric dimethylarginine (ADMA), blocks the synthesis of NO by the inhibition of the NOS active site. ADMA is a naturally occurring amino acid found in plasma and various tissues. An enhanced production of ADMA has been associated with impaired endothelial function in humans [68]. A possible role for this compound has been proposed in the physiological process of aging, as a positive correlation has been reported in healthy subjects between the plasmatic levels of ADMA and age [69]. Moreover, the effect ADMA has on accelerating endothelial cells senescence has been previously described [70].

### 4.3. Lower Expression and/or Activity of eNOS

Several studies have evaluated the expression of the enzyme eNOS in arteries of aged animals, but the results obtained have not provided definitive conclusions. While some studies, performed in smooth muscle cells, showed increased eNOS protein levels in the aorta and mesenteric arteries [71], others have reported no significant changes in eNOS expression in the same cellular type [72]. In human endothelial cells from peripheral veins and the brachial artery, there were no changes in eNOS expression during the aging process [73]. The discrepancies found among the published results could be due to the use of different animal models, arterial types (in human or animals) and the difference in age between old and young groups; however, there is a greater consensus over the reduced activity of the eNOS enzyme in aging [74]. Also, eNOS is regulated at post-transcriptional level involving PI3 kinase/Akt-dependent phosphorylation at Ser117, resulting in an increase in NO production from endothelial cells. In aged animals, the PI3K/Akt pathway is diminished and eNOS gene expression and enzymatic activity levels decrease [75]. However, the involvement in this low rate of eNOS phosphorylation in human vascular aging needs to be confirmed.

## 5. Vascular Aging and Cellular Senescence

Considerable evidence indicates that an imbalance between the magnitude of vascular injury and the capacity for repair appears to play an important role in age-related impaired endothelial function. These impairments are attributed to a decrease in the number and function of endothelial progenitor cells (EPCs) and/or impaired cell replication.

### 5.1. Number and Function of Endothelial Progenitor Cells (EPCs)

Accumulating evidence suggests that circulating bone marrow-derived EPCs contribute to vascular repair and regeneration, accelerating re-endothelialization and protecting against the initiation and progression of atherosclerosis [76]. EPCs express markers of both hematopoietic stem cells and endothelial cells on their surface and represent a very small subset of mononuclear cells, between 0.002% and 0.01% in peripheral blood and 0.2%–1% in umbilical cord blood [77]. Circulating EPC levels reflect vascular repair capacity, so that a reduction in the number of circulating EPCs predicts the occurrence of cardiovascular events [76]. In this situation, EPCs are mobilized from the bone marrow into circulation, reaching vascular injury areas where they are able to contribute to new blood vessel formation. Several studies have demonstrated that circulating EPCs are subject to changes associated with aging, which negatively affect their number and/or function. Consistent with this notion, the number of EPCs in healthy individuals is reduced with age [78]. Also, EPC mobilization is significantly impaired in older individuals compared with younger subjects [79] and the function of EPCs from older individuals also appears to be disrupted, according to *in vitro* studies [80]. Moreover, flow-mediated vasodilation was significantly correlated with the number of circulating EPCs in patients who had varying degrees of cardiovascular risk (but no history of cardiovascular disease) [81].

There is a wide range of environmental factors which influence EPC generation and function. Previous studies demonstrated that human EPCs express high levels of antioxidant enzymes as compared to mature endothelial cells [82]. Aging produces an impairment of their antioxidant capacity, reducing levels and activity of antioxidant enzymes such as glutathione peroxidase-1 [82].

Aging is also known to be associated with the development of chronic low grade inflammation, which contributes to impaired EPC function [56]. Deregulation of the pro-inflammatory cytokine TNF-α has been associated with the pathogenesis of atherosclerosis. Indeed, vascular aging is associated with upregulation of TNF-α, which can induce premature senescence in highly proliferative EPCs [83]. A decrease in EPC mobilization and function may be related to the reduced capacity of the aging endothelium to generate NO and the increased production of ROS [84]. The accumulation of oxidized low-density lipoprotein (oxLDL) with age also contributes to a reduction in the number of circulating EPCs, due to its inhibitory effect on eNOS expression and activity [85]. Another factor involved in EPC regeneration during aging is endothelial senescence, a process through which the EPC vascular repair mechanisms are damaged. The therapeutic use of the introduction of telomerase into EPCs has been shown to extend lifespan and improve vasculogenesis of these cells [86].

### 5.2. Impaired Cell Replication and Telomere Shortening in Aged Arteries

Telomeres are repeats of DNA–protein complexes, located at the ends of chromosomes, and are essential to the stability of chromosome and cell replication. The formation, maintenance and repair of telomeres are performed by the enzyme telomerase, but as a consequence of semiconservative DNA replication, the extreme terminals of chromosomes are not duplicated completely, causing successive shortening of the telomeres with each round of cell division [87]. Telomere length is regulated by pro-inflammatory cytokines and oxidative stress [88,89]: the latter promotes telomere shortening during cell replication *in vitro* and stimulates the synthesis of pro-inflammatory cytokines [20]. There is evidence of a correlation between ROS levels and the rate of telomere shortening. These studies suggest that an increase in intracellular ROS level could lead to an acceleration in the rate of telomere shortening. The progressive shortening of telomeres leads to senescence, apoptotic cell death, or the oncogenic transformation of somatic cells in various tissues. Telomere length, which can be affected by various lifestyle factors, may determine overall health, lifespan, and the rate at which an individual ages [90].

As a normal cellular process, telomere length decreases with age [91]. Recent studies in rodents have shown a causal effect of telomerase deficiency and telomere shortening on healthy aging and premature mortality [92,93]. Similarly, the length of telomeres isolated from endothelial cells of human arteries shows a strong inverse correlation with age [88]. The endothelial cell senescence observed during the normal aging process seems to be accelerated in the diseases associated with aging and in particular CVD [94].

### 5.3. Microparticles

Microparticles (MPs) are small fragments or vesicles from different types of cells (endothelial cells, platelets and leukocites) which are released during cell apoptosis, inflammatory activation and cellular stress [95]. They are made up of material from their cells of origin, which is a general characteristic of MPs [96]. MPs are present in plasma from healthy subjects, but their concentrations change in several clinical conditions. MP concentrations increase in patients with cardiovascular risk factors and after cardiovascular events [97]. Moreover, certain pharmacological treatments, used to treat cardiovascular diseases, reduce plasma MP concentrations. However, the pathophysiological effects of MPs *in vivo* are still poorly understood. Studies performed with MPs (*in vitro* or isolated *ex vivo*) have shown that they can influence some of the processes involved in atherogenesis, such as endothelial function, angiogenesis, inflammation and thrombosis, which suggests that MPs are not only markers, but they are also involved in cardiovascular diseases [98].

Several studies have reported an increase in MPs in aged diabetic rats [99] and an increase in leukocyte-derived MPs in aged mice with thrombosis [100]. In humans, there is an increase in platelet-derived MPs in the elderly compared to healthy individuals [101]. All these findings show the role of MPs in vascular pathology and suggest that, by themselves, they are the key to promoting premature vascular aging cellular senescence.

Recent studies have indicated that MPs have an effect on the endothelium through the increase in oxidative stress and endothelial inflammation, reducing the production of NO, or stimulating the platelet and macrophage adhesion to endothelial cells [102–104]. Burger *et al*. [104] have observed that MPs stimulate the production of ROS through NADPH oxidase [105], mitochondria, and NOS ciclooxigenase [106], inducing premature senescence of the endothelial cells. These results suggest that MPs contribute to the progression of vascular aging mechanism through a “feed-forward” method, where the increased formation of MPs from senescent endothelial cells also promotes cellular senescence through an increase in ROS production.

## 6. Mediterranean Diet: Does It Prevent Endothelial Aging?

The Mediterranean diet is a healthy diet which includes fish, vegetables, fruit, whole grains, legumes, olive oil, and less red meat and dairy products. Changes in lifestyle habits, such as diet and moderate exercise, can influence vascular repair mechanisms. Different studies have shown that a healthy diet and exercise induce a reduction in cell damage and endothelial dysfunction, both of which are factors responsible for reducing cardiovascular risk in the elderly [24,107].

Several intervention studies have suggested that the consumption of flavonoid-rich foods such as tea, red wine [108], cocoa and soya can improve endothelial function in patients with manifest cardiovascular and cerebrovascular disease [109,110]. In this way, Perez-Martinez *et al*., showed that the consumption of a Mediterranean diet reduced postprandial levels of oxidative stress biomarkers such as lipid peroxide, protein carbonyl, SOD activity and plasma H_2_O_2_ compared to a saturated fat-rich diet in metabolic syndrome subjects [38]. Similarly, this diet significantly attenuated the postprandial inflammatory state, including NF-κB, metalloproteinase-9 and tumor necrosis factor-α [33,39]. In addition, consumption of a Mediterranean diet and exercise led to a greater decrease in blood pressure and a greater increase in the number of EPC compared with the same diet without exercise [37].

Antioxidant supplementation can play an important role in delaying or reducing many of the adverse effects of aging. In this regard, it is interesting to review the beneficial effects that can be obtained through nutrition in the prevention of the deleterious effects induced by oxidative stress. The repair or prevention effects have been attributed to the presence of antioxidants, mainly contained in plant foods such as fruit, vegetables, whole grains, nuts and seeds [29,111,112]. Several studies indicate that vitamin E supplementation can improve antioxidant activity of cell membranes in elderly subjects [113,114]. Also, antioxidants such as the polyphenolic compound have anti-aging properties. In addition, a recent randomized controlled trial has shown that omega-3-polyunsaturated fatty acid supplementation lowered the concentration of serum pro-inflammatory cytokines [30]. On the other hand, Csiszar *et al*. indicated the possibility that supplementation of resveratrol, a diet-derived polyphenol, may confer significant vasoprotection in elderly humans [45,115,116].

Similarly, studies performed with elderly people have demonstrated that the consumption of a Mediterranean diet produced an increase in NO bioavailability, with a consequent improvement in endothelium-dependent endothelial function [25]. It is also associated with an improvement in endothelial regeneration capacity, producing an increased number of circulating EPCs and lower levels of microparticles, compared with the consumption of a saturated fatty acid-rich diet and a low-fat and high-carbohydrate diet, enriched with α-linolelic acid [35,37].

An improvement in lifestyle habits is also positively correlated with telomere length. In fact, telomere length has been associated with nutritional status in both human and animal models (Table 1). A healthy lifestyle, with a diet high in fruit and vegetables combined with exercise, lower body mass and not smoking is associated with longer telomeres [34]. Another study suggests that lower n-6:n-3 PUFA ratios can influence cell aging, increasing telomere length with a reduction of the n-6:n-3 ratio in a diet supplemented with omega-3 (n-3) PUFA [31]. A protein-restricted diet produced an increase in lifespan and a reduction in growth rate in rats, but also, the increased lifespan in such animals was associated with significantly longer telomeres in the kidney [117]. Cassidy *et al.* [40] demonstrated that leukocyte telomere length in women was positively correlated with dietary intake of fiber and negatively associated with dietary intake of polyunsaturated fatty acids, especially linoleic acid. Recent studies have shown that, in patients with coronary artery disease, there was an inverse relationship between baseline blood levels of marine omega-e fatty acids and the rate of telomere shortening [41]. Similarly, there is evidence to show the effect of the quality and quantity of dietary fat on telomere length, depending on the degree of oxidative stress that these diets produce. Therefore, the consumption of a saturated fatty acid-rich diet or a carbohydrate-rich diet induces telomere attrition, as a result of cell replication, which can be accelerated by the presence of increased oxidative stress [36]. However, the consumption of a Mediterranean type diet (monounsaturated fat-rich diet), rich in virgin olive oil, improves this profile and leads to a reduction in the degree of oxidative stress [24,25] and a decrease in the rate of telomere shortening [35]. Antioxidants can potentially protect telomeric DNA from oxidative damage caused by extrinsic and intrinsic DNA damaging agents, so a diet lacking antioxidants led to shorter telomeres, whereas consumption of an antioxidant rich diet such as vitamin C, E and β-carotene was associated with longer telomeres [35,118].

## 7. Conclusions and Future Expectations

Healthy dietary habits and moderate physical exercise improve endothelial dysfunction and oxidative stress, which are two factors involved in cardiovascular alterations associated with vascular aging. The consumption of a Mediterranean diet improves endothelial regenerative capacity as a result of a balance between cellular damage and repair. The mechanisms involved in this process can be associated with a decreased release of free radicals and a reduction in oxidative stress, due to the protective effects of both the monounsaturated fat and antioxidants present in this diet. Moreover, a Mediterranean diet may protect against endothelial cell senescence, generating a decrease in intracellular oxidative stress, telomere shortening and cellular apoptosis. All these mechanisms may be involved in an increased lifespan and a lower incidence of the diseases associated with aging present in populations which consume a Mediterranean-type diet.

These findings show that cellular oxidative stress, one of the major sources of damage in the dysregulation of biological systems, is closely linked to the generation of ROS and this can contribute to the development of cellular senescence, which leads to accelerated aging of the organism [119]. Senescent endothelial cells play a more important role morphologically and functionally in the development of atherosclerosis than normal cells. Epidemiological data indicate that young subjects with early signs of vascular senescence have an increased risk of developing CVD, so this suggests that the prevention or delay of the aging process could be used as a prophylaxis of vascular aging.

Antioxidant supplementation can play an important role in delaying or reducing many of the adverse effects of aging. Its repair or prevention effects have been attributed to the presence of antioxidants, mainly contained in plant foods such as fruit, vegetables, whole grains, nuts and seeds. Several studies have indicated that vitamin E and/or coenzyme Q_10_ supplementation can improve antioxidant activity of cell membranes in the elderly [24,25,120].

Dietary fat may also modulate oxidative stress in human endothelial cells, and a Mediterranean diet may bring considerable health benefits. Esposito *et al*. [121] have shown that in overweight or obese men, a prolonged adherence to a Mediterranean-style diet with or without caloric restriction, is associated with amelioration of multiple risk factors including a better cardiovascular risk profile and a reduced level of oxidative stress, all of which are markers of aging. Several researchers have suggested that the Mediterranean diet has a protective effect against cardiovascular, metabolic, cancer and other age-related diseases and degenerative diseases [24,25,122,123]. The Mediterranean dietary pattern is characterized by a high intake of fruit and vegetables, olive oil as the main source of fat intake, a low consumption of meat products, and moderate wine consumption. Visioli *et al.* [124] have linked the benefits of the Mediterranean diet with the protective role of phenolic compounds present in this type of diet, leading to a reduction of oxidative stress. Recent studies performed in the elderly have demonstrated that the Mediterranean diet protected endothelial cells against oxidative stress and prevented the development of cell senescence [35]. This study has shown that the consumption of a Mediterranean diet induces lower intracellular oxidative stress by decreasing levels of ROS. Therefore, this diet could modulate intracellular oxidative stress in the elderly, possibly due to minor components with antioxidant properties included in the Mediterranean diet. Recent studies confirm that excessive oxidative stress on endothelial cells promotes apoptosis [125]. Thus, the Mediterranean diet and/or the micronutrients present in this diet may participate in the regulation of these pathways, leading to lower apoptosis in endothelial cells [35].

In summary, dietary intervention, and particularly a Mediterranean-style diet, improves vascular dysfunction and microcirculation, and can play a role in the protection against the chronic diseases related to aging.

## Figures and Tables

**Table 1:** Effect of dietary nutrients and caloric restriction on cellular damage associated with aging.

Human Study	Mechanisms involved	Authors’ conclusions
Landberg *et al.*	Endothelial dysfunction	Beneficial effects of dietary compounds, fruit, vegetables, fish and nuts, on endothelial dysfunction [29]
Kiecolt-Glaser, J.K. *et al.*	Telomere length and Inflammation	The lower n-6: n-3 (polyunsaturated fatty acid) PUFA ratios may be beneficial for slowing biological aging [30,31]
Weiss, E.P. *et al.*	Vascular aging	Studies in animals and humans indicate that caloric restriction prevent many of the age-related changes in the structure and function of the cardiovascular system [32]
Scoditti, E. *et al*.	Inflammation	Mediterranean diet polyphenols suppressed inflammatory angiogenesis [33]
Mirabello, L. *et al.*	Telomere length	A healthy lifestyle with a diet high in fruit and vegetables combined with exercise, lower body mass and not smoking is associated with longer telomeres [34]
Marin, C. *et al.*	Endothelial progenitor cell, microparticles, oxidative stress and telomere length	The Mediterranean diet is associated with improvement in endothelial regeneration capacity, increased number of circulating endothelial progenitors cell (EPC), lower levels of microparticles, reduce oxidative stress and decreased telomere shortening rate [35,36]
Fernandez, J.M. *et al.*	Endothelial progenitor cell	The consumption of a Mediterranean diet and exercise led a greater decrease in blood pressure and a greater increase in EPC number [37]
Martinez, P. *et al.*	Markers of oxidative stress	The Mediterranean diet reduces postprandial levels of oxidative stress biomarkers such as lipid peroxide, protein carbonyl, superoxide dismutase (SOD) activity and plasma H_2_O_2_ [38]
Cruz-Teno, C. *et al.*	Inflammatory state	The Mediterranean diet attenuates the postprandial inflammatory state, including nuclear transcription factor-kappa B (NF-κB), metalloproteinase-9 and tumor necrosis factor-α [39]
Cassidy, A. *et al.*	Telomere length	The dietary intake of fiber is positively correlated with leukocyte telomere length in women and negatively associated with dietary intake of polyunsaturated fatty acids, especially linoleic acid [40]
Farzaneh-Far, E. *et al.* Kielcolt-Glaser, J.K. *et al.*	Telomere shortening	In patients with coronary artery disease, there was an inverse relationship between baseline blood levels of marine omega-3 fatty acids and the rate of telomere shortening [31,41]
Yubero-Serrano, E.M. *et al.*	Oxidative stress	The Mediterranean diet, rich in virgin olive oil, induced a reduction in the degree of oxidative stress. In addition, coenzyme Q10 supplementation can improve antioxidant activity of cell membranes in the elderly [24,25]
**Animal model study**	**Mechanisms involved**	**Author’s conclusion**
Jung, K.J. *et al*.	Inflammation	Caloric restriction appears to attenuate vascular NF-κB induction and endothelial activation in aged rats[42,43]
McCarty, M.F.	Nitric oxide production	A low-fat, whole-food, vegan diet or exercise training would be expected to decrease the risk of common age-related diseases [26]
***In vitro* study**	**Mechanisms involved**	**Author’s conclusion**
Csiszar, A. *et al*.	Mitochondria	Resveratrol induces mitochondrial biogenesis in cultured endothelial cells and in endothelia of mice with accelerated vascular aging [22,27]
Csiszar, A. *et al*. and Ungvari, Z. *et al*.	Inflammation and oxidative stress	*In vitro* studies suggest that the molecular mechanisms of resveratrol-mediated vasoprotection involve an inhibition of NF-κB and an upregulation of endothelial nitric oxide synthase (eNOS) and antioxidant enzymes [28,44–46]
Tang, Y. *et al.*	Cellular senescence and oxidative stress	In vitro studies suggest that resveratrol protects vascular cell senescence reducing the production of reactive oxygen species (ROS) [23,28]
